# Ovine *KRT81* Variants and Their Influence on Selected Wool Traits of Commercial Value

**DOI:** 10.3390/genes15060681

**Published:** 2024-05-24

**Authors:** Wenhao Li, Lingrong Bai, Huitong Zhou, Zhihe Zhang, Zhijie Ma, Guofang Wu, Yuzhu Luo, Jasmine Tanner, Jon G. H. Hickford

**Affiliations:** 1 Plateau Livestock Genetic Resources Protection and Innovative Utilization Key Laboratory of Qinghai Province, Key Laboratory of Animal Genetics and Breeding on Tibetan Plateau, Ministry of Agriculture and Rural Affairs, Qinghai Academy of Animal Science and Veterinary Medicine, Qinghai University, Xining 810016, China; qhdxlwh@163.com (W.L.); zhi-jiema@126.com (Z.M.); qhdxwuguofang@126.com (G.W.); 2 International Wool Research Institute/Faculty of Animal Science and Technology, Gansu Agricultural University, Lanzhou 730070, China; huitong.zhou@lincoln.ac.nz (H.Z.); 18205610566@163.com (Z.Z.); luoyz@gsau.edu.cn (Y.L.); 3 Gene-Marker Laboratory, Faculty of Agricultural and Life Sciences, Lincoln University, Lincoln 7647, New Zealand; lingrong.bai@lincolnuni.ac.nz (L.B.); jasmine.tanner@lincoln.ac.nz (J.T.)

**Keywords:** keratin K81, *KRT81*, polymorphism, wool trait, sheep

## Abstract

Keratins are the main structural protein components of wool fibres, and variation in them and their genes (*KRTs*) is thought to influence wool structure and characteristics. The PCR–single strand conformation polymorphism technique has been used previously to investigate genetic variation in selected coding and intron regions of the type II sheep keratin gene *KRT81*, but no variation was identified. In this study, we used the same technique to explore the 5′ untranslated region of *KRT81* and detected three sequence variants (*A*, *B* and *C*) that contain four single nucleotide polymorphisms. Among the 389 Merino × Southdown cross sheep investigated, variant *B* was linked to a reduction in clean fleece weight, while *C* was associated with an increase in both greasy fleece weight and clean fleece weight. No discernible effects on staple length or mean-fibre-diameter-related traits were observed. These findings suggest that variation in ovine *KRT81* might influence wool growth by changing the density of wool follicles in the skin, the density of individual fibres, or the area of the skin producing fibre, as opposed to changing the rate of extrusion of fibres or their diameter.

## 1. Introduction

Wool is primarily made up of two types of protein: keratins and keratin-associated proteins (KAPs). Wool keratins are the principal structural components of the fibre and form heterodimeric pairs that are then assembled into structures called intermediate filaments (IFs). These are embedded in, and covalently linked to, a diverse protein matrix composed of KAPs [1]. Two types of wool keratin have been defined: type I (acidic) and type II (basic-neutral) keratins. In sheep, a total of seventeen wool keratin genes (*KRTs*) have been identified, and these encompass ten type I wool keratin genes (*KRT31*, *KRT32*, *KRT33A*, *KRT33B*, *KRT34-KRT36*, *KRT38-KRT40*) and seven type II wool keratin genes (*KRT81-KRT87*) [2,3,4,5].

Research into genetic variation in wool *KRTs* is crucial for understanding their role in determining wool traits. Studies have been conducted on five type I wool *KRTs*: *KRT31* [6], *KRT33A* [5], *KRT34* [7], *KRT36* [8] and *KRT38* [8], as well as five type II wool *KRTs*: *KRT81* [8], *KRT83* [9], *KRT84* [10], *KRT85* [8] and *KRT87* [11]. To date, sequence variation has been observed in all the wool *KRTs* studied, with one exception. Sulayman et al. [8] used PCR–single strand conformation polymorphism (PCR-SSCP) to examine two exons and two introns of *KRT81* in Chinese Merino sheep, but no sequence variation was detected in these gene regions. This suggests a need to further investigate *KRT81*.

In this study, an investigation was conducted of the 5′ untranslated region (UTR) of ovine *KRT81* in Merino × Southdown cross sheep to ascertain if genetic variation existed, and if identified, to explore whether it affected selected wool traits that determine the commercial value of wool. This cross was being developed to obtain lower mean fibre diameter (MFD) and higher mean fibre curvature (MFC) wool in sheep that have faster liveweight gains, earlier maturation and better carcass meat yield. The overall aim was to obtain further insight into the genetic basis of variation in wool characteristics and potentially lay a foundation for the selective breeding of sheep to improve wool quality.

## 2. Materials and Methods

### 2.1. Sheep Blood and Wool Samples

Three hundred and eighty-nine Merino × Southdown cross sheep, these being the offspring of six sires, were investigated. These sheep were produced over several years. The sheep were of a similar age, and they were managed as part of a single mob on improved pasture. For each sheep, a venous blood sample from the ear was gathered onto TFN paper (Munktell Filter AB, Falun, Sweden), and genomic DNA that was bound to the paper was refined using a procedure described by Zhou et al. [12].

Wool samples were collected from the mid-side of the sheep at their first shearing at 12 months of age. At shearing, the weight of the fleece collected, known as the greasy fleece weight (GFW; kg), was recorded. Subsequently, various wool traits were measured on these samples by the NZ Wool Testing Authority Ltd. (Ahuriri, Napier, NZ) using International Wool Textile Organisation (IWTO; https://iwto.org/)-endorsed testing methods (https://iwto.org/resources/wool-testing-resources/), including wool yield (yield; %), MFD (µm), fibre diameter standard deviation (FDSD; µm), coefficient of variation of fibre diameter (CVFD, %), mean staple length (MSL; mm), MFC (°/mm), mean staple strength (MSS; N/ktex) and prickle factor (PF; %). The clean fleece weight (CFW; kg) was calculated from the GFW and yield values (CFW = yield/100 × GFW).

### 2.2. PCR–Single Strand Conformational Polymorphism (PCR-SSCP) Analysis

Two PCR primers were designed, based on an ovine *KRT81* gene sequence X62509 [3], to amplify a 427 bp fragment of the 5′ UTR region. The sequences of these primers were 5′-TGCACACACACAGGTCACC-3′ (forward primer) and 5′-GAATCCTGATCCGCAGGTC-3′ (reverse primer), and they were synthesised by Integrated DNA Technologies (Coralville, IA, USA). PCR amplification was conducted in a 15 µL reaction comprising the purified genomic DNA on a 1.2 mm punch of TFN paper, 0.25 µM of each primer, 150 µM of each dNTP (Bioline, London, UK), 2.5 mM of Mg^2+^, 0.5 U of Taq DNA polymerase (Qiagen, Hilden, Germany) and the 1× reaction buffer provided with the enzyme. The thermal profile consisted of an initial denaturation for 2 min at 94 °C, followed by 35 cycles of 30 s at 94 °C, 30 s at 62 °C and 30 s at 72 °C, and with an ultimate extension stage of 5 min at 72 °C. The thermal cycling was accomplished in S1000 thermal cyclers (Bio-Rad, Hercules, CA, USA).

A 1 μL aliquot of the PCR products was mixed with 7 μL of loading dye (98% formamide, 10 mM EDTA, 0.025% bromophenol blue, 0.025% xylene-cyanol) and after denaturation at 90 °C for 5 min, the samples were swiftly chilled on wet ice and then loaded on 16 × 18 cm, 12% polyacrylamide (acrylamide:bisacrylamide—37.5:1) gels containing 1% glycerol. Electrophoresis was performed for 16 h at 260 V and 24 °C in 0.5× TBE buffer, and the gels were silver-stained using the method of Byun et al. [13].

### 2.3. DNA Sequencing and Sequence Analyses

Representative selections of the PCR amplicons that displayed apparent homozygosity for different variants upon PCR-SSCP analysis were subjected to direct sequencing in both the forward and reverse directions at the Lincoln University DNA Sequencing Facility, NZ. In the situation where a variant was only observed in heterozygous sheep, a different sequencing method described by Gong et al. [14] was employed. In this approach, a PCR-SSCP band corresponding to the variant was removed as a gel slice from the polyacrylamide gel, crushed, and then used as a template for re-amplification. The resulting ‘homozygous’ amplicon was then subject to DNA sequencing.

Sequence alignments were carried out using DNAMAN (version 5.2.10, Lynnon BioSoft, Vaudreuil, Canada).

### 2.4. Statistical Analyses

Statistical analyses were conducted using Minitab version 16 (Minitab Inc., State College, PA, USA). General linear models (GLMs) were used to assess the impact of the presence or absence of the *KRT81* variants on the various wool traits that were measured. Genotypes with a frequency greater than 5% were used in GLMs to compare the various wool traits in sheep with those genotypes. To address the issue of undertaking multiple comparisons and reduce the chances of obtaining false positive results, a Bonferroni correction was applied and a post hoc Benjamini–Hochburg procedure was used to ascertain the potential for type I errors (false positives).

The models incorporated sire, gender and birth rank as fixed effects. Sire was identified to have an influence on all the wool traits, while gender and birth rank (whether the sheep was born as a single, twin or triplet) were identified as factors impacting only some wool traits. While the year of wool sample collection was also recorded, sire and year were absolutely confounded, with sire being chosen as the explanatory factor for the models as it explained more variation in the traits. The presence/absence model was: Y_jklm_ = µ + V_j_ + G_k_ + S_l_ + B_m_ + e_jklm_; where Y_jklm_ is the observed trait in the jklm^th^ animal, µ is the group raw mean for the trait, V_j_ is the effect of the j^th^ variant (presence and absence), G_k_ is the effect of gender, S_l_ is the effect of the l^th^ sire, B_m_ is the birth rank, and e_jklm_ is the random residual effect. The genotype model was: Y_jklm_ = µ + GT_j_ + G_k_ + S_l_ + B_m_ + e_jklm_; where Y_jkml_ is the observed trait in jklm^th^ animal, µ is the group raw mean for the trait, GT_j_ is the fixed effect of the j^th^ genotype, G_k_ is the effect of gender, S_l_ is the effect of the l^th^ sire, B_m_ is the birth rank, and e_jklm_ is the random residual effect.

## 3. Results

**Figure 1:**
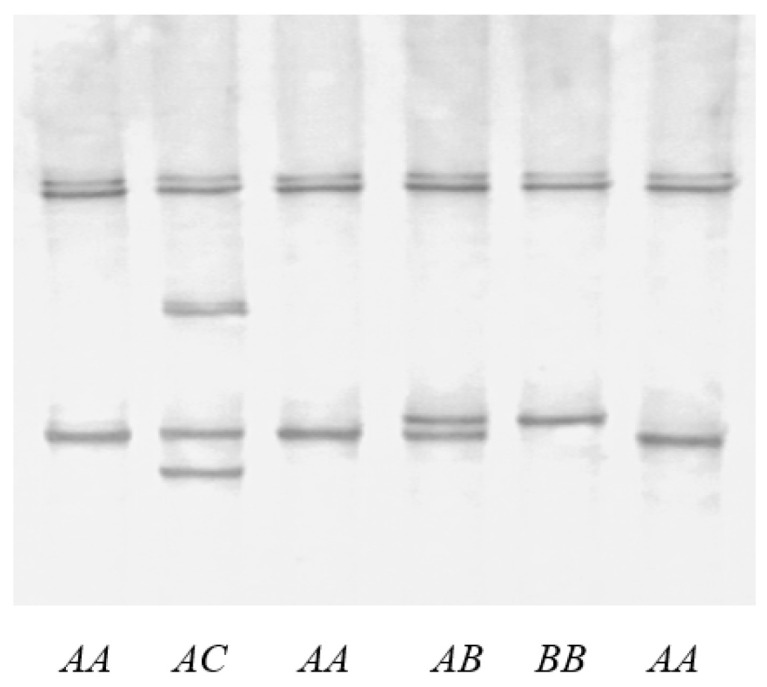
PCR-SSCP gel electrophoresis patterns for a fragment of the 5′ UTR of ovine *KRT81*. Three different patterns (*A*, *B* and *C*) are observed in either homozygous or heterozygous forms.

**Figure 2:**
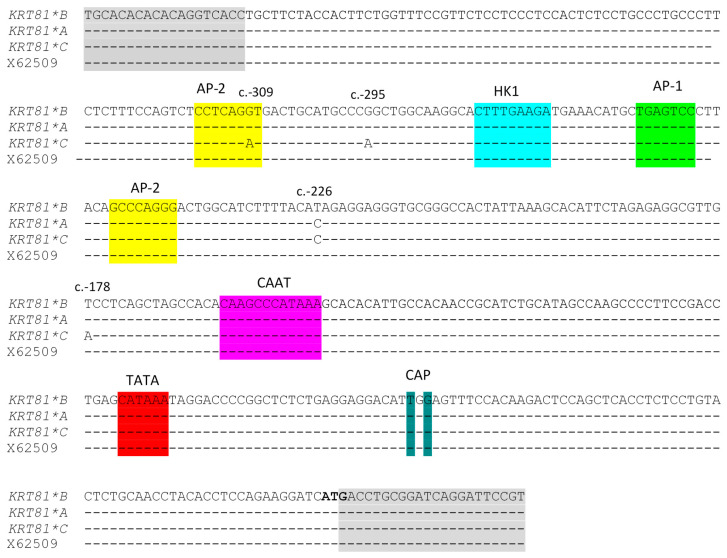
Alignment of the ovine *KRT81* sequences. Three variant sequences (*A*, *B* and *C*) identified in this study are aligned with the GenBank sequence X62509. The putative HK1, AP-1, AP-2, CAAT, TATA and two CAP sites identified by Powell et al. [3] are marked, and the start codon is highlighted in bold. Nucleotides identical to the top sequence are denoted by dashes. Grey shaded regions indicate the PCR primer biding sites. The positions of the SNPs identified are indicated above the sequences.

Three different SSCP banding patterns were identified for the 5′ UTR amplicon of ovine *KRT81* (Figure 1). Sequencing of selected amplicons revealed three sequence variants that were named *A*, *B*, and *C*. Upon comparing these sequence variants, four SNPs were identified as c.-309G/A, c.-295G/A, c.-226T/C and c.-178T/A (Figure 2). The sequence of variant *B* was identical to the reference gene sequence X62509.

Four genotypes out of the six that might be expected were detected in the 389 sheep investigated. These genotypes and their frequencies were: *AA* (31.9%), *AB* (40.6%), *AC* (10.0%) and *BB* (17.5%). Consequently, the frequency of variants *A*, *B*, and *C* in this population was 57.2%, 37.8%, and 5.0%, respectively.

The variant presence/absence models revealed that the variation in *KRT81* was associated with two wool traits, GFW and CFW. Specifically, the presence of *B* was associated with a decrease in CFW, while the presence of variant *C* was linked to an increase in both GFW and CFW (Table 1). As might possibly be expected given the relationship between CFW and GFW, there was a trend suggesting an association between *KRT81* variation and yield. No associations were observed with other wool traits.

The corrected genotype models also revealed a difference in GFW and CFW between genotypes. These two associations persisted upon post hoc Benjamini–Hochburg analysis at a false discovery rate of 25%. Genotype *AC* was found to be associated with higher GFW and CFW, whereas genotype *AB* exhibited lower GFW and CFW (Table 2). Once again, there was a trend suggesting a relationship between genotype and yield, while no associations were observed with the other wool traits.

## 4. Discussion

The identification of four SNPs comprising three sequence variants in the 5′ UTR of ovine *KRT81* is noteworthy given the absence of sequence variation in the coding and intron regions described in a previous study [8]. This 5′ UTR region putatively contains several sequence motifs identified by Powell et al. [3], such as HK1, AP-1 AP-2, TATA, CAAT and CAP sites (Figure 2). While Powell et al. [3] used a primer extension assay and suggested that the presence of two putative CAP sites at c.-65 and c.-63, an online tool (https://www.fruitfly.org/cgi-bin/seq_tools/promoter.pl; accessed on 22 February 2024), predicts a putative CAP site at c.-67, which is different to those proposed by Powell et al. [3]. Interestingly, Powell et al. [3] identified a CAAT sequence motif (5′-CAAGCCCATAAA-3′), which significantly differs from the consensus sequence 5′-GG(T/C)CAATCT-3′ [15]. However, no sequence resembling the CAAT consensus sequence was found by us in the region analysed.

One of the SNPS (c.-309G/A) identified in this study is in an AP-2 binding site identified by Powell et al. [3], although the sequence they report (5′-CCTCAGGT-3′) is dissimilar to the ovine AP-2 binding sequence (5′-CCCCAGGGC-3′) reported in the ovine placental lactogen gene by Limesand and Russell [16].

These sequence motifs may play a role in regulating wool keratin gene expression, and although most of the SNPs revealed in this study are not located within these identifiable sequence motifs, variation in these regions could still exert an influence on gene expression by altering promoter structure. Regardless, the variation revealed may have a functional consequence and impact the structural and functional characteristics of wool fibres.

In this respect, two type I wool keratin genes *KRT31* and *KRT34*, have also been reported to be polymorphic in their 5′ UTR regions, and this variation has also been associated with variation in key wool traits [6,7], although another study failed to find variation in the type II wool keratin gene *KRT83* promoter [9]. This variation, along with other reports of variation in wool keratins [7,8,17,18] and the KAP genes [19,20,21,22], suggests that genetic variation exists in nearly all wool protein genes. The polymorphic nature of these genes, combined with the extensive number of genes that have been identified, suggests considerable complexity underpinning the variation in wool fibres and wool traits.

The finding that variation in ovine *KRT81* affects the two related fleece weight traits, without impacting staple length and fibre diameter traits has not been observed for other *KRTs* and *KRTAPs*. For example, variation in *KRT31* [6], *KRTAP1-2* [23] and *KRTAP20-1* [24] affects fleece weight traits, but also other traits like MSL and/or the fibre diameter traits, like MFD, FDSD and CVFD. The absence of an effect on MSL or fibre diameter traits leaves three things that may affect the weight of the fleece: variation in the density of the individual fibres, variation in the number of wool follicles per unit area of skin, or variation in the amount of skin that contains follicles, the latter suggesting *KRT81* is in some way affecting the skin, not just the wool follicles therein. Given that Yu et al. [10] illustrated that *KRT81* was expressed in the cortex of the wool follicle, the latter two seem less likely, though not impossible; thus, individual fibre density appears most likely to be what the variation in the promotor of *KRT81* may be affecting. The level of expression of the gene may play a role in determining the quantity of heterodimers produced for the assembly into intermediate filaments, with this in turn influencing the ratio of intermediate filaments to the matrix, and this potentially affecting fibre density. This is speculative and further research is most certainly needed to gain a better understanding of whether the 5′ UTR variation revealed here affects transcription and gene expression, and subsequently wool traits.

## 5. Conclusions

This study identified three sequence variants of ovine *KRT81* and reported four SNPs in the 5′ untranslated region. The variation in this region was found to be associated with wool fleece weights but not with staple length or mean-fibre-diameter-related traits, suggesting that the gene influences wool growth, likely by affecting the density of individual fibres.

## Figures and Tables

**Table 1:** Association of *KRT81* variants with various wool traits.

Trait ^1^	Variant ^2^	Mean ± SE ^3^		*p* ^4^
		Present	Absent	
GFW (kg)	*A*	2.3 ± 0.03	2.5 ± 0.05	0.061
	*B*	2.4 ± 0.03	2.4 ± 0.04	0.187
	*C*	2.6 ± 0.07	2.4 ± 0.03	**0.006**
CFW (kg)	*A*	1.7 ± 0.02	1.8 ± 0.04	0.363
	*B*	1.7 ± 0.03	1.8 ± 0.03	**0.045**
	*C*	1.9 ± 0.05	1.7 ± 0.02	**0.023**
Yield (%)	*A*	72.8 ± 0.44	71.4 ± 0.79	0.081
	*B*	72.1 ± 0.50	73.2 ± 0.54	0.067
	*C*	72.3 ± 1.00	72.6 ± 0.44	0.777
MSL (mm)	*A*	83.5 ± 1.00	82.0 ± 1.71	0.413
	*B*	82.9 ± 1.11	83.6 ± 1.19	0.590
	*C*	83.0 ± 2.18	83.2 ± 0.97	0.899
MSS (N/ktex)	*A*	23.6 ± 0.55	23.2 ± 0.98	0.753
	*B*	23.3 ± 0.62	23.6 ± 0.68	0.751
	*C*	23.8 ± 1.24	23.4 ± 0.55	0.793
MFD (µm)	*A*	19.5 ± 0.15	19.7 ± 0.25	0.442
	*B*	19.6 ± 0.16	19.4 ± 0.17	0.237
	*C*	19.8 ± 0.32	19.5 ± 0.14	0.303
FDSD (µm)	*A*	4.2 ± 0.05	4.1 ± 0.08	0.380
	*B*	4.1 ± 0.05	4.1 ± 0.06	0.975
	*C*	4.3 ± 0.11	4.1 ± 0.05	0.118
CVFD (%)	*A*	21.0 ± 0.19	20.6 ± 0.33	0.173
	*B*	21.2 ± 0.19	21.3 ± 0.20	0.563
	*C*	21.8 ± 0.38	21.2 ± 0.16	0.131
MFC (^o^/mm)	*A*	87.9 ± 1.08	89.5 ± 1.91	0.398
	*B*	89.0 ± 1.22	87.0 ± 1.32	0.190
	*C*	86.4 ± 2.44	88.4 ± 1.07	0.435
PF (%)	*A*	2.5 ± 0.24	2.3 ± 0.42	0.690
	*B*	2.5 ± 0.27	2.4 ± 0.29	0.658
	*C*	3.5 ± 0.58	2.5 ± 0.26	0.102

^1^ GFW—greasy fleece weight; CFW—clean fleece weight; MSL—mean staple length; MSS—mean staple strength; MFD—mean fibre diameter; FDSD—fibre diameter standard deviation; CVFD—coefficient of variation of fibre diameter; MFC—mean fibre curvature; PF—prickle factor. ^2^ Variant *A* was present in 321 sheep and absent in 68 sheep, variant *B* was present in 226 sheep and absent in 163 sheep, and variant *C* was present in 39 sheep and absent in 350 sheep. ^3^ Predicted means and standard errors of those means derived from GLMs. ^4^ *p* < 0.05 are highlighted in bold.

**Table 2:** The effect of *KRT81* genotypes on various wool traits.

Trait ^1^		Mean ± SE ^2^			*p* ^3^
	*AA* (n = 124)	*AB* (n = 158)	*AC* (n = 39)	*BB* (n = 68)	
GFW (kg)	2.2 ± 0.10 ^ab^	2.2 ± 0.10 ^b^	2.4 ± 0.11 ^a^	2.3 ± 0.10 ^a^	**0.004**
CFW (kg)	1.7 ± 0.08 ^ab^	1.6 ± 0.08 ^b^	1.8 ± 0.09 ^a^	1.7 ± 0.09 ^ab^	**0.031**
Yield (%)	76.4 ± 1.55	75.3 ± 1.51	75.4 ± 1.66	74.2 ± 1.63	0.095
MSL (mm)	85.5 ± 3.02	85.2 ± 2.93	84.4 ± 3.23	83.9 ± 3.17	0.354
MSS (N/ktex)	23.8 ± 1.93	23.6 ± 1.88	23.7 ± 2.07	23.1 ± 2.03	0.932
MFD (µm)	19.1 ± 0.46	19.3 ± 0.45	19.7 ± 0.49	19.4 ± 0.48	0.354
FDSD (µm)	4.0 ± 0.16	4.11 ± 0.16	4.3 ± 0.17	4.0 ± 0.17	0.163
CVFD (%)	21.0 ± 0.59	21.2 ± 0.57	21.7 ± 0.63	20.7 ± 0.61	0.166
MFC (°/mm)	88.8 ± 3.78	90.2 ± 3.67	87.8 ± 4.04	91.5 ± 3.96	0.520
PF (%)	1.7 ± 0.84	2.3 ± 0.82	3.2 ± 0.90	2.0 ± 0.88	0.113

^1^ GFW—greasy fleece weight; CFW—clean fleece weight; MFD—mean fibre diameter; FDSD—fibre diameter standard deviation; CVFD—coefficient of variation of fibre diameter; MSL—mean staple length; MSS—mean staple strength; MFC—mean fibre curvature; PF—prickle factor. ^2^ Estimated marginal means, standard errors and *p* values derived from GLMs. Means within rows that do not share a superscript letter (e.g., a) were different at *p* < 0.05. ^3^ *p* < 0.05 are highlighted in bold.

## Data Availability

The original data used in this paper are available by contacting the corresponding author upon request.

## References

[1] Powell B.C., Rogers G.E. (1997). The role of keratin proteins and their genes in the growth, structure and properties of hair. EXS.

[2] Wilson B.W., Edwards K.J., Sleigh M.J., Byrne C.R., Ward K.A. (1988). Complete sequence of a type-I microfibrillar wool keratin gene. Gene.

[3] Powell B., Crocker L., Rogers G. (1992). Hair follicle differentiation: Expression, structure and evolutionary conservation of the hair type II keratin intermediate filament gene family. Development.

[4] Powell B.C., Crocker L.A., Rogers G.E. (1993). Complete sequence of a hair-like intermediate filament type II keratin gene. DNA Seq..

[5] Yu Z., Wildermoth J.E., Wallace O.A., Gordon S.W., Maqbool N.J., Maclean P.H., Nixon A.J., Pearson A.J. (2011). Annotation of sheep keratin intermediate filament genes and their patterns of expression. Exp. Dermatol..

[6] Chai W., Zhou H., Gong H., Wang J., Luo Y., Hickford J.G. (2019). Nucleotide variation in the ovine *KRT31* promoter region and its association with variation in wool traits in Merino-cross lambs. J. Agric. Sci..

[7] Chai W., Zhou H., Gong H., Hickford J.G. (2022). Variation in the ovine *KRT34* promoter region affects wool traits. Small Rumin. Res..

[8] Sulayman A., Tursun M., Sulaiman Y., Huang X., Tian K., Tian Y., Xu X., Fu X., Mamat A., Tulafu H. (2018). Association analysis of polymorphisms in six keratin genes with wool traits in sheep. Asian-Australas. J. Anim. Sci..

[9] Chai W., Zhou H., Forrest R.H.J., Gong H., Hodge S., Hickford J.G.H. (2017). Polymorphism of *KRT83* and its association with selected wool traits in Merino-cross lambs. Small Rumin. Res..

[10] Yu Z., Gordon S.W., Nixon A.J., Bawden C.S., Rogers M.A., Wildermoth J.E., Maqbool N.J., Pearson A.J. (2009). Expression patterns of keratin intermediate filament and keratin associated protein genes in wool follicles. Differentiation.

[11] Yu Z.D., Gordon S.W., Wildermoth E.E., Wallance O.A.M., Nicon A.J., Pearson A.J. Identification of novel wool keratin intermediate filament genes in sheep skin. Proceedings of the New Zealand Society of Animal Production.

[12] Zhou H., Hickford J.G.H., Fang Q. (2006). A two-step procedure for extracting genomic DNA from dried blood spots on filter paper for polymerase chain reaction amplification. Anal. Biochem..

[13] Byun S.O., Fang Q., Zhou H., Hickford J.G.H. (2009). An effective method for silver-staining DNA in large numbers of polyacrylamide gels. Anal. Biochem..

[14] Gong H., Zhou H., Plowman J.E., Dyer J.M., Hickford J.G. (2010). Analysis of variation in the ovine ultra-high sulphur keratin-associated protein KAP5-4 gene using PCR-SSCP technique. Electrophoresis.

[15] Breathnach R., Chambon P. (1981). Organization and expression of eucaryotic split genes coding for proteins. Annu. Rev. Biochem..

[16] Limesand S., Anthony R.V. (2001). Novel activator protein-2α splice-variants function as transactivators of the ovine placemtal lactogen gene. Eur. J. Biochem..

[17] Yu X., Li S., Zhou H., Zhao F., Hu J., Wang J., Liu X., Li M., Zhao Z., Hao Z. (2024). Spatiotemporal expression and haplotypes identification of *KRT84* gene and their association with wool traits in Gansu Alpine fine-wool sheep. Genes.

[18] Singh H., Gahlot G.C., Narula H.K., Pannu U., Ashraf M., Chopra A. (2023). Polymorphism of keratin intermediate filament type I gene and its association with wool quality traits in Magra sheep. Indian J. Small Rumin..

[19] Zhou H., Gong H., Wang J., Luo Y., Li S., Tao J., Hickford J.G. (2021). The complexity of the ovine and caprine keratin-associated protein genes. Int. J. Mol. Sci..

[20] Kumar R., Meena A.S., Chopra A., Kumar A. (2020). Keratin gene expression differences in wool follicles and sequence diversity of high glycine-tyrosine keratin-associated proteins (KAPs) in Magra sheep of India. J. Nat. Fibers.

[21] Zhou H., Li W., Bai L., Wang J., Luo Y., Li S., Hickford J.G. (2023). Ovine *KRTAP36-2*: A new keratin-associated protein gene related to variation in wool yield. Genes.

[22] Gong H., Zhou H., Hodge S., Dyer J.M., Hickford J.G. (2015). Association of wool traits with variation in the ovine KAP1-2 gene in Merino cross lambs. Small Rumin. Res..

[23] Sallam A.M., Gad-Allah A.A., Albetar E.M. (2022). Genetic variation in the ovine KAP22-1 gene and its effect on wool traits in Egyptian sheep. Arch. Anim. Breed..

[24] Gong H., Zhou H., Bai L., Li W., Li S., Wang J., Luo Y., Hickford J.G. (2019). Associations between variation in the ovine high glycine-tyrosine keratin-associated protein gene *KRTAP20-1* and wool traits. J. Anim. Sci..

